# Severe Poisoning by Homatropin Used as an Eye Instillation

**Published:** 1910-10-29

**Authors:** 


### Toxicology.

### POISONING BY HOMATROPIN USED AS AN EYE INSTILLATION.

Homatropin is generally regarded as so safe a drug to use for purposes of dilating the pupil that it is employed in all sorts of cases without a thought as to any possible dangers that may arise from it. So much less is the duration of pupillary paralysis after it than after atropine sulphate that homatropin has practically replaced atropine for the ordinary purposes of eye-testing in adults. Nevertheless it is important to know that even quite severe symptoms have been known to follow its use, whilst milder inconveniences are not very uncommon. The following are the notes of a case in which the toxic effects were for the time being very grave.

The patient was a woman, aged 26. She was ordered to instil two drops of a 2 per cent. solution of hydrobromate of homatropin into the eyes every five minutes for half an hour. After the second instillation she felt dizzy and faint, and soon afterwards she was obliged to send for her doctor, who found her in a highly nervous state, with dizziness and fulness of the head and much congestion of the face; the skin was hot and dry, the pupils were widely dilated, and the mucous membranes of the mouth and throat were so parched that swallowing was almost impossible. Breathing was rapid, and the pulse-rate was 130 per minute. Complete coma soon supervened, the temperature rose to 100° F., whilst the face developed a vivid red colour. Shortly afterwards the pulse became weak and the end of the fingers livid and cold. A hot saline enema was given; it was returned discoloured, but otherwise unaltered. The symptoms gradually grew less alarming; the coma was succeeded by a busy, talkative delirium, and the circulation improved, though the extremities were still livid and cold six hours after the instillations. After an anxious night the patient was quite rational next morning, though she was very depressed and too nauseated to take food. The pupils were still widely dilated. The urine was scanty and dark. The temperature was 99° F., the pulse-rate 76, and the respiration-rate 20 per minute. Steady improvement continued, although for several days there was extreme weakness, and the patient complained of a faint feeling about her heart. The pupils remained dilated for a week, and all these symptoms followed the instillation of no particularly large dose of homatropin instilled into the conjunctival sac in the ordinary way. So similar were the symptoms to those that have followed overdoses of atropine that a doubt might have arisen as to whether or not some mistake might perhaps have been made, but the possibility of the substitution of atropine for homatropin was disproved by analysis of the solution actually used by the patient.

